# Effect of *Clostridium butyricum* Supplementation on *in vitro* Rumen Fermentation and Microbiota With High Grain Substrate Varying With Media pH Levels

**DOI:** 10.3389/fmicb.2022.912042

**Published:** 2022-06-23

**Authors:** Peixin Jiao, Ziwei Wang, Xin Wang, Yanan Zuo, Yuqing Yang, Guanghui Hu, Changming Lu, Xiaolai Xie, Li Wang, Wenzhu Yang

**Affiliations:** ^1^College of Animal Science and Technology, Northeast Agricultural University, Harbin, China; ^2^Hubei Greensnow Biological Technology Co., Ltd., Xianning, China; ^3^Lethbridge Research and Development Centre, Lethbridge, AB, Canada

**Keywords:** batch culture, *Clostridium butyricum*, media pH, microbiota, rumen fermentation

## Abstract

*Clostridium butyricum* (*C. butyricum*) can survive at low pH, and it has been widely used as an alternative to antibiotics for the improvement of feed efficiency and animal health in monogastrics. A recent study suggested that the improved ruminal fermentation with supplementing *C. butyricum* is may be associated with increasing the abundance of rumen microbiota in Holstein heifers, as ruminal pH plays a key role in rumen microbiota and the probiotics are often active in a dose-dependent manner. The objective of this study was to determine the effects of increasing the doses of *C. butyricum* on gas production (GP) kinetics, dry matter disappearance (DMD), fermentation characteristics, and rumen microbiota using a high grain substrate in batch culture varying with media pH levels. The doses of *C. butyricum* were supplemented at 0 (control), 0.5 × 10^6^, 1 × 10^6^, and 2 × 10^6^ CFU/bottle, respectively, at either media pH 6.0 or pH 6.6. The fermentation microbiota at 0 and 1 × 10^6^ CFU/bottle were determined using the 16S rRNA high throughput sequencing technology. Overall, the GP, DMD, total volatile fatty acid (VFA) concentration, and the ratio of acetate:propionate were higher (*P* <0.01) at media pH 6.6 than at pH 6.0. However, there was interaction between pH × dose of C. butyricum for rate constant of GP (*P* = 0.01), average GP rate (*P* = 0.07), and volume of GP (*P* = 0.06); with the increase in *C. butyricum* supplementation, the GP kinetics were not changed at media pH 6.0, but the volume (*P* = 0.02), rate of GP (*P* = 0.01), and average GP rate (*P* = 0.01) were quadratically changed at media pH 6.6. The DMD was not affected by increasing the supplementation of C. butyricum. The molar proportions of propionate (*P* <0.09), butyrate (*P* <0.06), and NH_3_-N concentration (*P* = 0.02) were quadratically changed with increasing supplementation of *C. butyricum* regardless of media pH levels. The interactions between media pH level and dose of *C. butyricum* supplementation were noticed for alpha diversity indexes of Shannon (*P* = 0.02) and Evenness (*P* = 0.04). The alpha diversity indexes increased (*P* <0.05) except for Chao1 with supplementation of *C. butyricum*. The unweighted uniFrac analysis showed that the group of control at media pH 6.0 and control at media pH 6.6, and supplementation of *C. butyricum* and control at media pH 6.0 clustered separately from each other. At the phylum level, relative abundance (RA) of *Bacteroidota* was lower (*P* <0.01) and *Firmicutes* was higher (*P* <0.01) at media pH 6.6 than pH 6.0. Moreover, RA of *Proteobacteria* decreased (*P* <0.05) with supplemented *C. butyricum* at either media pH 6.6 or pH 6.0. At media pH 6.6, RA of *Rikenellaceae_RC9_gut_group* and *Prevotella* were decreased, and CAG-352 was increased (at genus level) compared to pH 6.0. Supplementation of *C. butyricum* decreased RA of *Rikenellaceae_RC9_gut_group* and increased *CAG-352* at media pH 6.0. It could hence be concluded that manipulating media pH level and supplementation of *C. butyricum* effectively modulated *in vitro* rumen fermentation characteristics and microbiota but in a dose depending manner of *C. butyricum* addition.

## Introduction

Probiotics are defined as live microbials and are widely used in livestock animals to increase feed digestion and improve growth performance and health status (Ohashi and Ushida, 2009; Kober et al., 2022). The bacteria-based probiotics were originally developed mainly for their intestinal activities; whereas, studies show some indications that some bacterial probiotics may also have positive effects on the rumen, especially helping to reduce the incidence of ruminal acidosis (Krehbiel et al., 2003). Ramaswami et al. (2005) reported that adding lactic acid bacteria probiotics could help to establish the indigenous microbial population in rumen. This bacterium also improves the equilibrium among ruminal microbial groups (Bae et al., 2003). The probiotics with different bacteria species such as *Lactobacillus* and *Enterococcus* help prevent ruminal acidosis, by allowing the microorganisms to adapt to the presence of lactate in the rumen (Yoon and Stern, 1995; Ghorbani et al., 2002). Several modes of action of bacteria-based probiotics in the rumen were suggested, such as an interaction of bacterial strains with rumen microorganisms to improve rumen fermentation and inhabitation in detrimental microorganisms by producing some antimicrobial substances like bacteriocins (McAllister et al., 2011). With the growing trend of the ban of antibiotics all over the world due to the problems of antibiotic residues and environmental pollution, probiotics have been extensively paid attention to as an economically competitive alternative to antibiotics in ruminants. Although, probiotics have been widely used to improve growth performance and production of ruminants (Ban and Guan, 2021), to our best knowledge, the effects of probiotics varied with studies. Our previous studies reported that the inconsistency of responses to probiotics with lactic acid bacteria depended on several factors, including media pH level, strains, and dose of probiotics or type of substrate incubated (Jiao et al., 2017, 2018).

*Clostridium butyricum* (*C. butyricum*) is a gram-positive anaerobe that can produce butyric acid and form spores, which can survive in low pH and high bile concentrations, and exists in the intestines of healthy animals and humans (Juan et al., 2017). To date, *C. butyricum* has been widely used as an alternative to antibiotics in the improvement of growth performance, feed efficiency, and health in monogastrics, such as poultry (Wang et al., 2020) or pigs (Zhang et al., 2020). The possible mode of action may be attributed to the increase of secretion and activities of digestive enzymes in the intestine through the modulation of gut microbial composition or its production of butyric acid (Liu et al., 2017; Duan et al., 2018). In ruminants, a recent study suggested that *C. butyricum* inclusion improved ruminal fermentation and growth performance by increasing the abundance of rumen microbiota and molar proportion of propionate and butyrate of Holstein heifers (Li et al., 2021).

Ruminal pH plays a key role in fermentation pattern and microbiota, and it is primarily affected by diet composition; for example, with high grain-fed animals, ruminal pH can be reduced below the optimum for fibrolytic microbial population (Yang et al., 2002; He et al., 2014). Because the fibrolytic microorganisms are particularly sensitive to low pH (Russell and Wilson, 1996), ruminal microbial activities are often compromised and rumen fermentation patterns are changed to less acetate production in high grain-fed animals (Jiao et al., 2019). However, it is not known whether the response of supplementation of *C. butyricum* varies with supplemental doses of strain and media pH level which are critical factors that could impact the probiotic activities in the rumen (Jiao et al., 2017). We hypothesized that supplementing *C. butyricum* would alter rumen microbiota, and thus affect rumen fermentation characteristics, and its activity will vary with media pH level and dose of strain. The objective of this study was to evaluate the effects of supplementation of *C. butyricum at* varying doses on gas production (GP) kinetics, fermentation characteristics, and microbiota in the batch culture at low (6.0) and high media pH (6.6).

## Materials and Methods

### *Clostridium butyricum* Samples and Substrate

The *C. butyricum* samples were provided by the Greensnow Biological Biotechnology Co., Ltd. (Wuhan, China), and its concentration was 2.0 × 10^8^ CFU/g. The substrate that was used in this study was a typical diet for lactation dairy cows in China, and it consisted of 40% forage and 60% concentrate mixture (dry matter basis; Table 1).

**Table 1:** Ingredients and chemical composition of experimental diets.

**Item**	**Content**
**Ingredients, %**	
Alfalfa hay	11.3
Alfalfa Silage	5.2
Corn Silage	22.2
Steam-flaked corn	14.7
Beet pulp	4.3
Soybean meal	13.6
Expanded soybean	3.1
Corn gluten meal	11.1
Double low rapeseed meal	3.8
Cottonseed meal	3.8
Sodium bicarbonate	0.9
Premix^a^	6.0
**Chemical composition, % of DM**	
DM, %	78.8
Organic matter	93.2
Crude protein	17.9
Ether extract	6.0
Neutral detergent fiber	32.3
Acid detergent fiber	16.5
Calcium	0.78
Phosphorus	0.39
Metabolic energy (MJ/kg)	8.7

a *Premix contained (per kilogram): 1,500 mg of Fe, 1,500 mg of Mn, 800 mg of Cu, 30 mg of I, 2,000 mg of Zn, 60 mg of Co, 80 mg of Se, 300,000 IU of VA, 100,000 IU of VD_3_, and 2,000 IU of VE*.

### Experimental Design and Inoculum

All experimental procedures associated with animals in the present study were performed according to the experimental license (protocol number: NEAU-[2011]-9) from Northeast Agricultural University (Harbin, China).

The *in vitro* experiment was a complete randomized design with two media pH levels (6.0 vs. 6.6) and four dosages of *C. butyricum* factorial arrangement of treatment. The two media pH levels were selected according to the continuous culture conducted by Yang et al. (2002). The four dosages of *C. butyricum* were 0 (control), 0.5 × 10^6^, 1 × 10^6^, and 2 × 10^6^ CFU/glass bottle, respectively, according to the manufacturer's recommendations. Two ruminally fistulated lactation dairy cows fed a diet consisting of 400 (g/kg DM) forage and 600 concentrate (DM basis) were used as rumen inoculum donors. A total mixed ration was prepared daily and offered twice daily at 0600 and 1,800 h.

### *In vitro* Batch Culture

Rumen contents were collected from various locations within the rumen 2 h after the morning feeding, composited, and immediately squeezed using a nylon mesh. The strained rumen fluid was kept in an insulated, air-tight container, and then immediately transferred to the laboratory and warmed in a water bath at 39°C. Before adding into the bottles, the rumen fluid was restrained through four layers of cheesecloth to remove particles. The pH of ruminal fluid was measured with a pH meter (B20PI, SympHony Benchtop Meters; VWR, Edmonton, AB, Canada) after straining and it was 6.62, 6.69, and 6.51, respectively, for the first, second, and third run.

Glass bottles (125 mL) with rubber stoppers and aluminum caps were used to perform batch cultures in this study. Filter bags (F57; Ankom Technology, Macedon, NY, USA) were washed in acetone solution and weighed after drying. The substrate (approximately 0.5 g DM) was ground to pass a 1-mm screen and weighed into the filter bags. The *C. butyricum* samples were pre-added to the bottles according to the supplemental doses. The high (6.6) and low (6.0) media pH were achieved by adjusting the volume of sodium bicarbonate in the buffer solution according to the method described by Jiao et al. (2019). At each media pH level, three additional bottles containing the same volume of buffer and rumen fluid without substrate or *C. butyricum* samples were used as blanks for correcting GP. The same culture was repeated in another two runs on different days.

Forty-five milliliters of freshly prepared buffer and 15 mL of strained rumen fluid were added into the bottles, and carbon dioxide was added to the bottles to remove the air in the headspace. Then, the bottles were sealed with rubber stoppers and aluminum caps, and then transferred to a shaking incubator (SPH-2102C, Shanghai huyueming Scientific Instrument Co., Ltd, Shanghai, China) at 39°C. Gas volume was detected at 3, 6, 9, 12, and 24 h during incubation using a pressure transducer (model HT-935, Hongcheng Technology, Shenzhen, China) with a 23-gauge needle (0.6 mm) through rubber stoppers. Pressure values, corrected for the amount of substrate DM incubated and the gas released from the blanks were used to generate gas volume (GV) using the equation of Romero-Perez and Beauchemin (2018): GV = 4.7047 × (gas pressure) + 0.0512 × (gas pressure^2^). Kinetics of GP were estimated by fitting GP data to an exponential model (Ørskov and McDonald, 1979): y = GV × (1 – e^−*c*×[*t*−*lag*]^), where “y” is the cumulative volume of gas produced at the time “t” (h), “GV” is the asymptotic GV, “c” is the constant fractional rate of GP, and “lag” is the time (h) between inoculation and commencement of GP. Average GP rate (AGPR, mL gas/h) between the start of the incubation and the time at which cumulative GP reached half of its asymptotic value was calculated using the equation of García-Martínez et al. (2005):


$$\begin{array}{l} \text{AGPR }=\;\left(\text{GV }\times \text{ c}\right)/\left[2\;\times \;\left(\text{Ln}2\;+\text{ c }\times \text{ Lag}\right)\right] \end{array}$$

After 24 h of incubation, the bottles were placed in cold water to stop fermentation. The aluminum caps and rubber stoppers were removed, and the pH of the fermentation liquid was measured using a portable pH meter (PHS-3C; Nanjing Nanda Analytical Instrument Application Research Institute, Nanjing, China). Five milliliters of fermentation liquid were collected from each bottle and preserved with 1 mL of metaphosphoric acid (25%, w/v) and sulfuric acid (1%, v/v), respectively, and stored at −20°C for the later analysis of volatile fatty acid (VFA) and NH_3_-N concentration. The three bottles of fermentation media with control and a medium level of *C. butyricum* were pooled by the run, respectively, and two samples of 5 mL of fermentation liquid were collected, immediately frozen in liquid nitrogen, and preserved at −80°C until DNA extraction.

### Microbial DNA Isolation and 16S RRNA High Throughput Sequencing

The analysis of the bacterial community in fermentation media was performed at LCSciences (Hangzhou, China). In brief, DNA from fermentation media was extracted using the E.Z.N.A. ®Stool DNA Kit (D4015, Omega, Inc., USA) according to the manufacturer's instructions. The PCR amplification of the bacterial 16S rRNA genes spanning the V4-V5 region (forward primer 515F: 5'-GTGCCAGCMGCCGCGGTAA-3'; reverse primer 907R: 5'-CCGTCAATTCMTTTRAGTTT-3') was determined. The 5' ends of the primers were tagged with specific barcodes per sample and sequencing universal primers. PCR amplification was performed in a total volume of 25 μL reaction mixture containing 25 ng of template DNA, 12.5 μL PCR Premix, 2.5 μL of each primer, and PCR-grade water to adjust the volume. The PCR conditions to amplify the prokaryotic 16S fragments consisted of an initial denaturation at 98°C for 30 s; 32 cycles of denaturation at 98°C for 10 s, annealing at 54°C for 30 s, and extension at 72°C for 45 s; and then final extension at 72°C for 10 min. The PCR products were confirmed with 2% agarose gel electrophoresis. Throughout the DNA extraction process, ultrapure water, instead of a sample solution, was used to exclude the possibility of false-positive PCR results as a negative control. The PCR products were purified by AMPure XT beads (Beckman Coulter Genomics, Danvers, USA) and quantified by Qubit (Invitrogen, USA). The amplicon pools were prepared for sequencing and the size and quantity of the amplicon library were assessed on Agilent 2100 Bioanalyzer (Agilent, USA) and with the Library Quantification Kit for Illumina (Kapa Biosciences, Woburn, USA), respectively. The libraries were sequenced on the NovaSeq PE250 platform.

Paired-end reads were assigned to samples based on their unique barcode and truncated by cutting off the barcode and primer sequence. Paired-end reads were merged using FLASH (version 1.2.8, http://ccb.jhu.edu/software/FLASH/). Quality filtering on the raw reads was performed under specific filtering conditions to obtain the high-quality clean tags according to the fqtrim (version 0.94, http://ccb.jhu.edu/software/fqtrim/). Chimeric sequences were filtered using Vsearch software (version 2.3.4, https://github.com/torognes/vsearch). Alpha diversity and beta diversity were calculated by normalized to the same sequences randomly. Then, according to SILVA (Release 138, https://www.arbsilva.de/documentation/release138/) classifier, feature abundance was normalized using the relative abundance of each sample. Alpha diversity is applied in analyzing the complexity of species diversity for a sample through five indices, including Chao1, OTU, Evenness, Shannon, Simpson, and all these indices in the samples were calculated with QIIME2 (https://qiime2.org/). Beta diversity was calculated by QIIME2. The blast was used for sequence alignment, and the feature sequences were annotated with the SILVA database for each representative sequence.

### Chemical Analyses

The substrate was analyzed for DM (method 930.15), ash and organic matter (method 942.05), crude protein (N × 6.25) (method 990.03), and acid detergent fiber was determined according to AOAC (2005; method 973.18) (AOAC, 2005). Neutral detergent fiber was analyzed as described by Van Soest et al. (1991) using heat-stable α-amylase and sodium sulfite. The fermentation VFA concentration was quantified using a gas chromatograph (GC-2010, Shimadzu) equipped with a capillary column (30 m × 0.32 mm i.d., 1-μm phase thickness, Zebron ZB-FAAP, Phenomenex, Torrance, CA, USA), and flame ionization detection and crotonic acid (trans-2-butenoic acid) were used as the internal standard (He et al., 2014). The NH_3_-N concentrations were analyzed according to the methods described by Broderick and Kang (1980).

### Statistical Analysis

Data were subjected to statistical analysis using the MIXED procedure of SAS (SAS Inst. Inc. Cary, NC) with the model including fixed effects of media pH, supplemental dose of *C. butyricum* and their interactions, and the random effects of run. Tukey's multiple comparison test was used to examine the significance of treatments. The CONTRAST statement of SAS with linear and quadratic orthogonal contrasts was used to determine the effect of increasing the dose of *C. butyricum*. Differences were declared significant at *P* ≤ 0.05. Trends were discussed at 0.05 < *P* ≤ 0.10 unless otherwise stated.

## Results

### Gas Production Kinetics and DMD

There was no interaction between media pH level and dose of *C. butyricum* supplementation on asymptotic GP and lag time, whereas the rate constant of GP (*P* = 0.01) and AGPR (trend, *P* = 0.07) interacted between the two factors (Table 2). Elevated media pH from 6.0 to 6.6 consistently increased (*P* <0.01) the GV and AGPR regardless of the dosages of *C. butyricum* supplementation. Increasing supplemental dose of *C. butyricum* did not change the GP kinetics at media pH 6.0, whereas quadratically changed asymptotic GP (*P* = 0.02) and rate constant of GP (*P* = 0.01) and AGPR (*P* = 0.01) at media pH 6.6. Supplementation of low *C. butyricum* had a greater (*P* <0.05) rate constant of GP than control, but a further increase of *C. butyricum* addition did not change the rate of GP. No significant interaction between media pH and the dose of *C. butyricum* on DMD was noticed. The DMD was consistently greater (*P* <0.01) with media pH 6.6 than pH 6.0. However, the DMD was not affected by increasing the dose of *C. butyricum* at either media pH level.

**Table 2:** Effects of media pH and supplemental doses of *C. butyricum* on gas production (GP) kinetics and dry matter disappearance (DMD).

	***Doses of C***.***butyricum***^**2**^					***P*** **-value** ^**3**^			
**Item^**1**^**	**Control**	**Low**	**Medium**	**High**	**SEM**	**pH**	**Treatment**	**Linear**	**Quadratic**
**pH6.0**									
GV, ml/g DM	156	152	158	155	4.0	<0.01	0.75	0.92	0.83
C, %/h	6.45	6.25	6.64	6.58	0.382	<0.01	0.60	0.45	0.99
Lag, h	2.01	2.35	2.17	2.42	0.167	0.86	0.33	0.17	0.77
AGPR, ml/h	6.11	5.65	6.29	5.99	0.298	<0.01	0.26	0.89	0.96
DMD, %	50.7	47.9	48.2	48.9	1.48	<0.01	0.58	0.51	0.19
**pH6.6**									
GV, ml/g DM	179	200	197	191	11.1	<0.01	0.07	0.39	0.02
C, %/h	7.55^b^	8.44^a^	7.95^ab^	7.71^b^	0.546	<0.01	0.01	0.76	0.01
Lag, h	2.13	2.26	2.43	2.27	0.732	0.86	0.24	0.31	0.08
AGPR, ml/h	7.77	9.80	9.09	8.47	1.375	<0.01	0.03	0.77	0.01
DMD, %	51.1	56.3	52.9	52.4	1.77	<0.01	0.09	0.91	0.11

a,b *Means within a row with different superscripts differ (P <0.05)*.

1 *GV, asymptotic gas volume; C, rate constant of GP; Lag, initial delay before GP begins (h); AGPR, average gas production rate*.

2 *Control, Low, Medium, and High were supplemented, respectively, with 0, 0.5 ×10^6^, 1 ×10^6^, and 2 ×10^6^ CFU/bottle of C. butyricum*.

3 *pH = pH 6.0 vs. 6.6; Linear, linear effect of C. butyricum addition; Quadratic, quadratic effect of butyricum addition; Interactions: GV: pH × Dose, P = 0.06; C: pH × Dose, P = 0.01; AGPR: pH × Dose, P = 0.07*.

### Fermentation Characteristics

There was no interaction of media pH level with the dose of *C. butyricum* on total VFA concentration and the molar proportion of individual VFA (Table 3). The increase of media pH increased (*P* <0.01) VFA concentration, but the total VFA concentration was not affected by increasing the dose of *C. butyricum*. Molar proportions of acetate and branch-chained VFA (BCVFA) were higher (*P* <0.01) and that of propionate was lower (*P* <0.01) at media pH 6.6 than pH 6.0. As a result, the ratio of acetate to propionate (A:P) was higher (*P* <0.01) at media pH 6.6 than at pH 6.0. Increasing the supplementation of *C. butyricum* did not alter the proportion of acetate, but it tended to quadratically change molar proportions of propionate at pH 6.0 (*P* = 0.06) and pH 6.6 (*P* = 0.09) or linearly (*P* <0.01) increased proportion of butyrate at pH 6.0, and quadratically (*P* = 0.06) at pH 6.6 without affecting A:P ratio at either media pH 6.6 or pH 6.0. The concentration of NH_3_-N was not affected by media pH level, but it linearly increased (*P* <0.01) with increasing the supplementation of *C. butyricum* at media pH 6.0 or 6.6 without interaction between media pH level and supplementation of *C. butyricum*.

**Table 3:** Effects of media pH and supplemental doses of *C. butyricum* on fermentation characteristics.

	***Doses of C***.***butyricum***^**2**^					***P*** **-value** ^**3**^			
**Item^**1**^**	**Control**	**Low**	**Medium**	**High**	**SEM**	**pH**	**Treatment**	**Linear**	**Quadratic**
**pH6.0**									
Total VFA, mM	55.1	54.7	58.8	57.1	4.38	<0.01	0.37	0.30	0.43
mol/100 mol									
Acetate (A)	50.1	49.3	49.8	48.8	0.55	<0.01	0.38	0.16	0.85
Propionate (P)	26.3	26.1	25.7	26.2	0.29	<0.01	0.19	0.64	0.06
Butyrate	14.4^b^	15.4^a^	15.7^a^	15.9^a^	0.59	<0.01	<0.01	<0.01	0.03
BCVFA	1.77	1.80	1.87	1.87	0.056	<0.01	0.51	0.20	0.52
A:P	1.88	1.89	1.94	1.87	0.031	<0.01	0.44	0.52	0.32
NH_3_-N, mM	14.0^b^	14.5^b^	15.7^a^	15.5^a^	0.64	0.54	<0.01	<0.01	<0.01
**pH6.6**									
Total VFA, mM	60.4	65.2	63.4	59.2	2.81	<0.01	0.39	0.50	0.15
mol/100 mol									
Acetate (A)	52.2	53.3	51.5	52.2	0.84	<0.01	0.14	0.53	0.82
Propionate (P)	23.9	23.1	23.5	23.7	0.41	<0.01	0.18	0.87	0.09
Butyrate	14.2^b^	14.2^b^	15.6^a^	14.8^ab^	0.38	<0.01	0.03	0.11	0.06
BCVFA	2.11	2.13	2.10	1.96	0.065	<0.01	0.19	0.06	0.30
A:P	2.19	2.30	2.20	2.21	0.047	<0.01	0.08	0.71	0.30
NH_3_-N, mM	13.6^c^	14.7^b^	15.8^a^	16.1^a^	0.76	0.54	<0.01	<0.01	0.02

a,b,c *Means within a row with different superscripts differ (P <0.05)*.

1 *VFA, volatile fatty acids; BCVFA, branched-chain VFA*.

2 *Control, Low, Medium, and High were supplemented, respectively, with 0, 0.5 × 10^6^, 1 × 10^6^, and 2 × 10^6^ CFU/bottle of C. butyricum*.

3 *pH = pH 6.0 vs. 6.6; T, comparison among control, low, medium, and high; L, linear effect of C. butyricum addition; Q, quadratic effect of butyricum addition; Interactions: acetate proportion, pH × Dose, P = 0.08*.

### Microbiome Diversity Analysis After 24 h of Incubation

A total of 1,852,493 valid reads were obtained, with an average of 77,187 sequences for each sample, and the ratio of valid data to raw data was 90.09% to 93.73% (Supplementary Table S1). The rarefaction curves on the number of OTUs indicated that all curves approached a plateau, indicating that the sampling depth was sufficient and adding more OTUs results without changing the slope of the curve (Supplementary Figure S1).

The alpha-diversity analysis showed that the index of Evenness was higher (*P* <0.01) at media pH 6.0 than at pH 6.6 after 24 h of fermentation (Table 4). There was no interaction between media pH and *C. butyricum* supplementation on the indexes of OUT number, Simpson, and Chao1; Supplementation of *C. butyricum* increased the indexes of OUT number (*P* = 0.05) and Simpson (*P* = 0.04). Interactions of pH with *C. butyricum* addition on indexes of Shannon (*P* = 0.02) and Evenness (*P* = 0.04) were observed; Supplementation of *C. butyricum* increased the indexes of Shannon (*P* = 0.01) and Evenness (*P* = 0.05).

**Table 4:** Effects of different supplemental doses of *C. butyricum* and media pH on diversity indices of ruminal fermentation fluid associated microbiome in *in vitro* fermentation.

	**Treatments** ^**1**^					***P*** **-value**		
	**pH6.0**		**pH6.6**					
**Index**	**Control**	**Medium**	**Control**	**Medium**	**SEM**	**pH**	**CB**	**pH × CB**
OTU	1,967	2,139	2,114	2,123	43.1	0.14	0.05	0.07
Shannon	9.44^b^	9.66^a^	9.48^b^	9.48^b^	0.041	0.09	0.01	0.02
Simpson	0.995	0.996	0.995	0.995	0.0002	0.19	0.04	0.06
Chao1	1,971	2,144	2,121	2,130	43.9	0.14	0.06	0.08
Evenness	0.863^b^	0.874^a^	0.858^b^	0.858^b^	0.0024	<0.01	0.05	0.04

a,b *Means within a row with different superscripts differ (P <0.05)*.

1 *Control and Medium were supplemented, respectively, with 0 and 1 ×10^6^ CFU/bottle of C. butyricum*.

**Figure 1:**
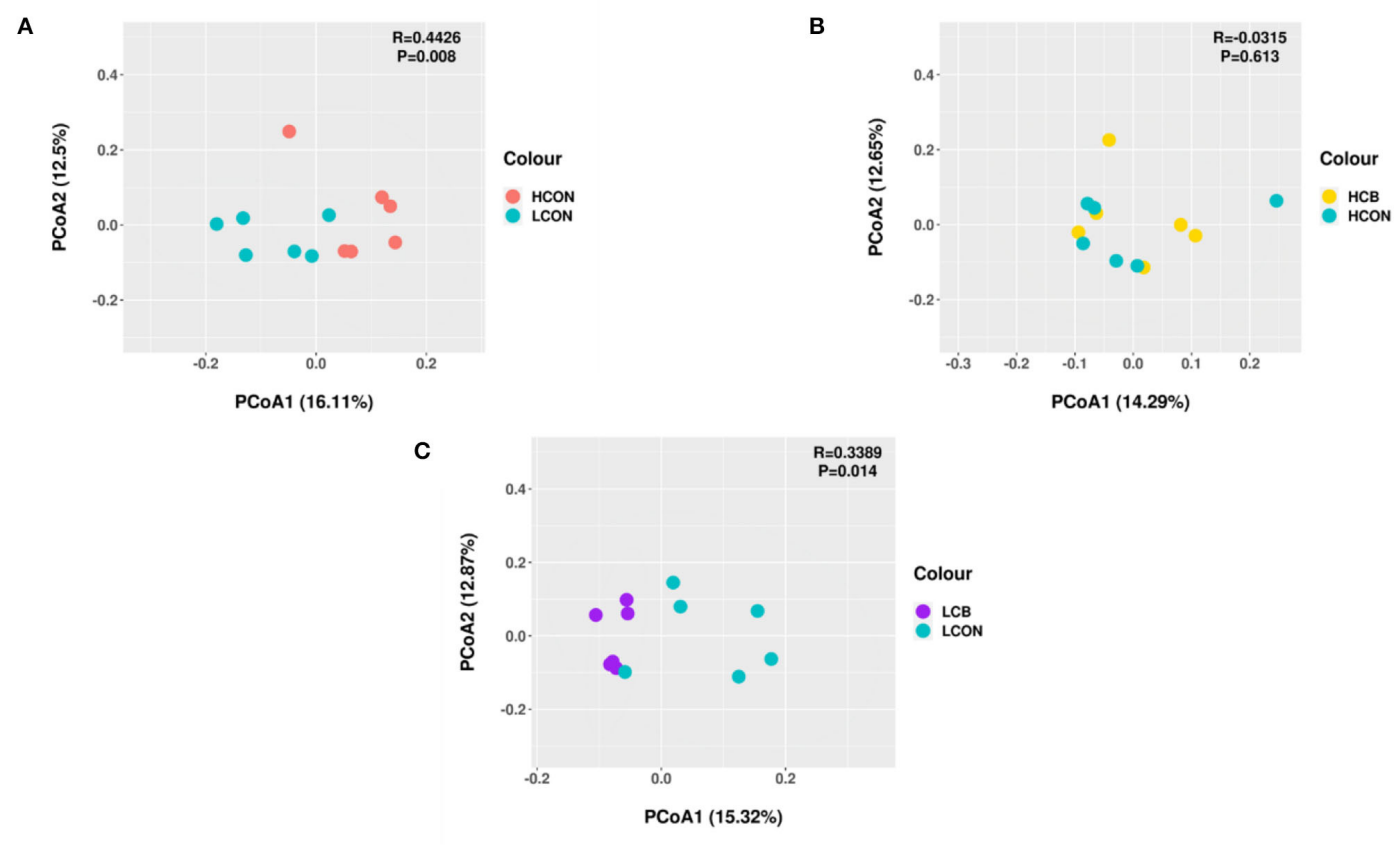
Principal coordinates analysis (PCoA) based on unweighted uniFrac distance matrices of ruminal fermentation fluid after 24 h of *in vitro* incubation. **(A)** HCON vs. LCON; **(B)** HCB vs. HCON; **(C)** LCB vs. LCON. HCON, control at media pH 6.6; LCON, control at media pH 6.0; HCB, *C. butyricum* supplemented at 1 ×10^6^ CFU/bottle at media pH 6.6; LCB, *C. butyricum* supplemented at 1 ×10^6^ CFU/bottle at media pH 6.0.

Principal coordinates analysis (PCoA) was conducted and illustrated based on unweighted uniFrac distance matrices to characterize the beta-diversity of the bacterial communities in ruminal fermentation media between two levels of pH without adding *C. butyricum*, or between control and *C. butyricum* at media pH 6.0 and media pH 6.6, respectively (Figure 1). The PCoA profile showed that the two groups of ruminal bacterial microbiota at media pH 6.0 and pH 6.6 were distinctly separated from each other (Figure 1A). At media pH 6.0, the ruminal bacterial microbiota supplemented with *C. butyricum* was distinctly separated from the control (Figure 1C). However, the clustering of bacterial microbiota at pH 6.6 from *C. butyricum* and control overlapped, and no clear distinction was noticed (Figure 1B). Moreover, the results of ANOSIM analysis showed a statistical difference in the composition of ruminal microbiota between media pH 6.6 and pH 6.0 without *C. butyricum* (*R* = 0.4462, *P* = 0.008) and *C. butyricum* and control at pH 6.0 (*R* = 0.3389, *P* = 0.014).

**Figure 2:**
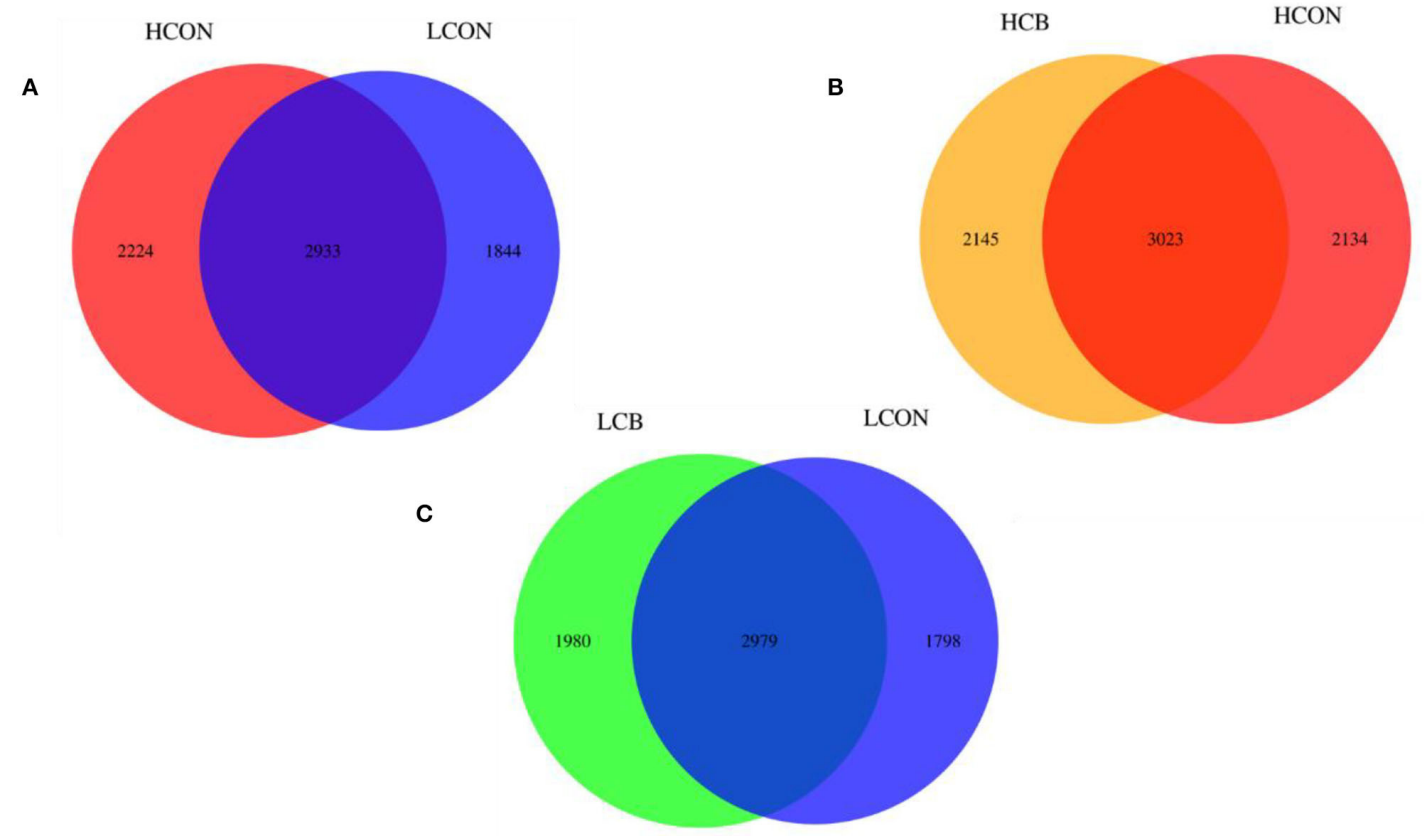
Analysis of OTUs' exclusiveness of microbiome of ruminal fermentation fluid after 24 h of *in vitro* incubation. **(A)** HCON vs. LCON; **(B)** HCB vs. HCON; **(C)** LCB vs. LCON. HCON, control at media pH 6.6; LCON, control at media pH 6.0; HCB, *C. butyricum* supplemented at 1 ×10^6^ CFU/bottle at media pH 6.6; LCB, *C. butyricum* supplemented at 1 ×10^6^ CFU/bottle at media pH 6.0.

The Venn diagram in the fermentation fluid showed that the two control groups of media pH6.6 and pH6.0 shared 2933 OTUs, and had 2224 and 1844 exclusive OTUs, respectively (Figure 2A). At media pH 6.6, the groups of *C. butyricum* and control shared 3023 OTUs, respectively, with 2145 and 2134 exclusive OTUs (Figure 2B). There were 2979 OTUs that were shared by *C. butyricum* and control at media pH 6.0, respectively, and 1980 and 1798 exclusive OTUs (Figure 2C).

### Relative Abundance of Microbiota After 24 h of Incubation

**Figure 3:**
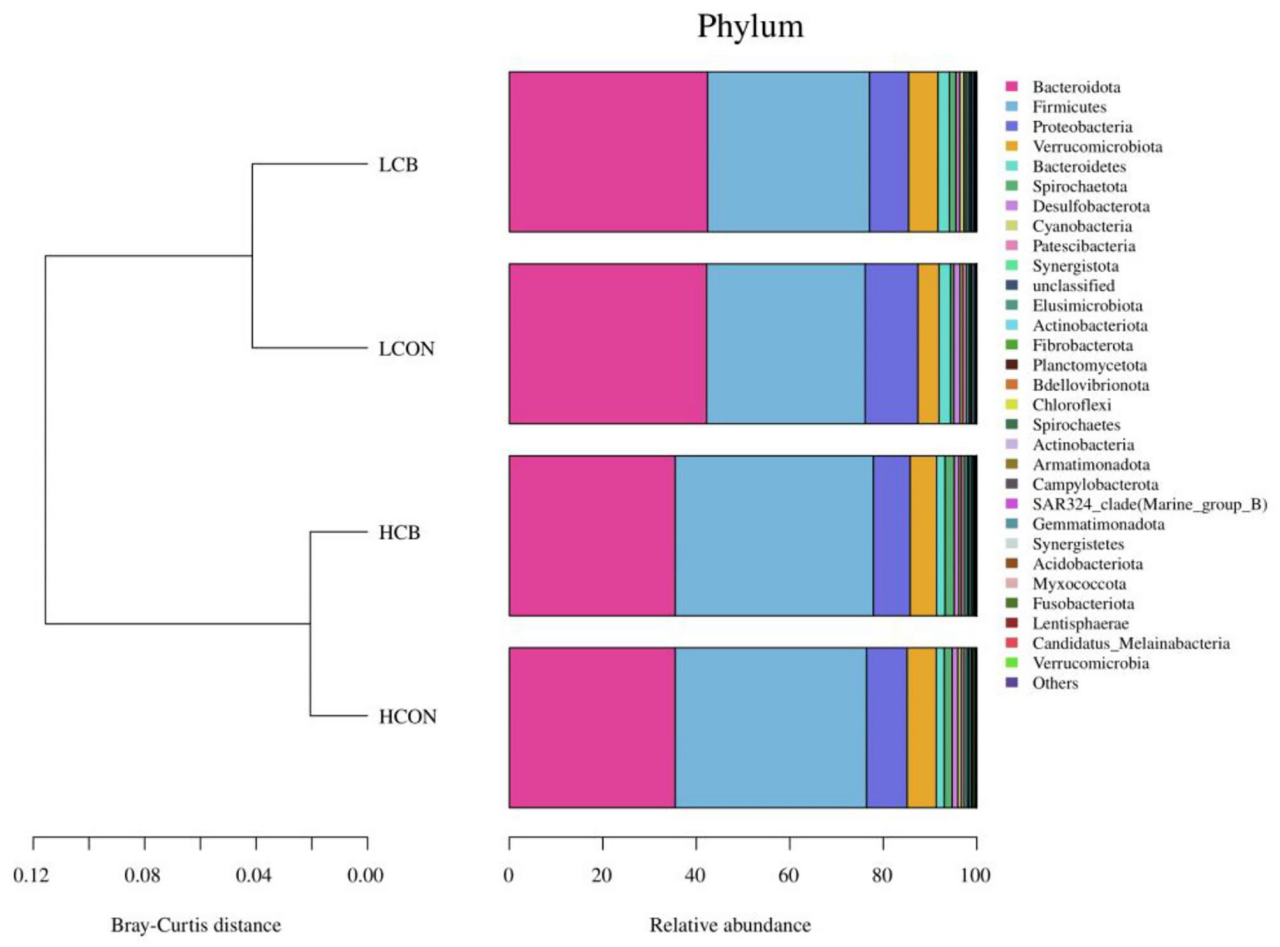
Top 30 relative abundance of the microbiota of ruminal fermentation fluid after 24 h of *in vitro* incubation that determined at the phylum level. HCON, control at media pH 6.6; LCON, control at media pH 6.0; HCB, *C. butyricum* supplemented at 1 ×10^6^ CFU/bottle at media pH 6.6; LCB, *C. butyricum* supplemented at 1 ×10^6^ CFU/bottle at media pH 6.0.

**Figure 4:**
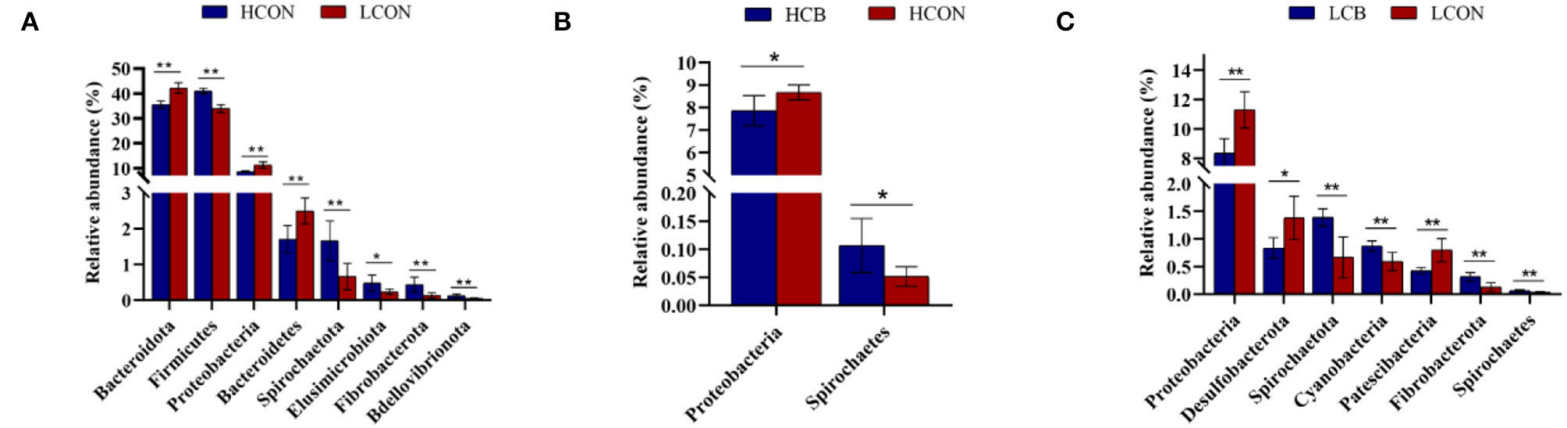
Relative abundance of microbiota in ruminal fermentation fluid that is significantly different between groups at the phylum level. **(A)** HCON vs. LCON; **(B)** HCB vs. HCON; **(C)** LCB vs. LCON. HCON, control at media pH 6.6; LCON, control at media pH 6.0; HCB, *C. butyricum* supplemented at 1 ×10^6^ CFU/bottle at media pH 6.6; LCB, *C. butyricum* supplemented at 1 ×10^6^ CFU/bottle at media pH 6.0. Values are shown as means ± standard errors. *P <0.05; **P <0.01.

The top 30 relative abundances (RA) of microbiota in fermentation fluid after 24 h of incubation were determined at the phylum level (Figure 3; Supplementary Figure S2). The *Bacteroidetes* and *Firmicutes* were the dominant microbiota in the ruminal microbial community regardless of media pH levels or whether *C. butyricum* was supplemented. The RAs of *Bacteroidetes* were 35.49, 35.49, 42.21, and 42.43%, respectively, and the RAs of *Firmicutes* were 40.94, 42.39, 33.89, and 34.63%, respectively, in groups of control, *C. butyricum* at pH 6.6, control and *C. butyricum* at pH 6.0. The RA of *Bacteroidota, Proteobacteria* and *Bacteroidetes* were lower (*P* <0.01), and *Firmicutes* (*P* <0.01), *Spirochaetota* (*P* <0.01), *Elusimicrobiota* (*P* <0.05), *Spirochaetes* (*P* <0.01), and *Bdellovibrionota* (*P* <0.01) were higher at media pH 6.6 than pH 6.0 (Figure 4A). At media pH 6.6, supplementation of *C. butyricum* decreased (*P* <0.05) the RA of *Proteobacteria* and increased (*P* <0.05) the *Spirochaetota* RA compared with control (Figure 4B). In comparison with control at pH 6.0, supplementation of *C. butyricum* decreased the RA of *Proteobacteria* (*P* <0.01), *Desulfobacterota* (*P* <0.05), and *Patescibacteria* (*P* <0.01), and increased *Spirochaetes* (*P* <0.01), *Cyanobacteria* (*P* <0.01), *Fibrobacterota* (*P* <0.01), and *Spirochaetes* (*P* <0.01) (Figure 4C).

**Figure 5:**
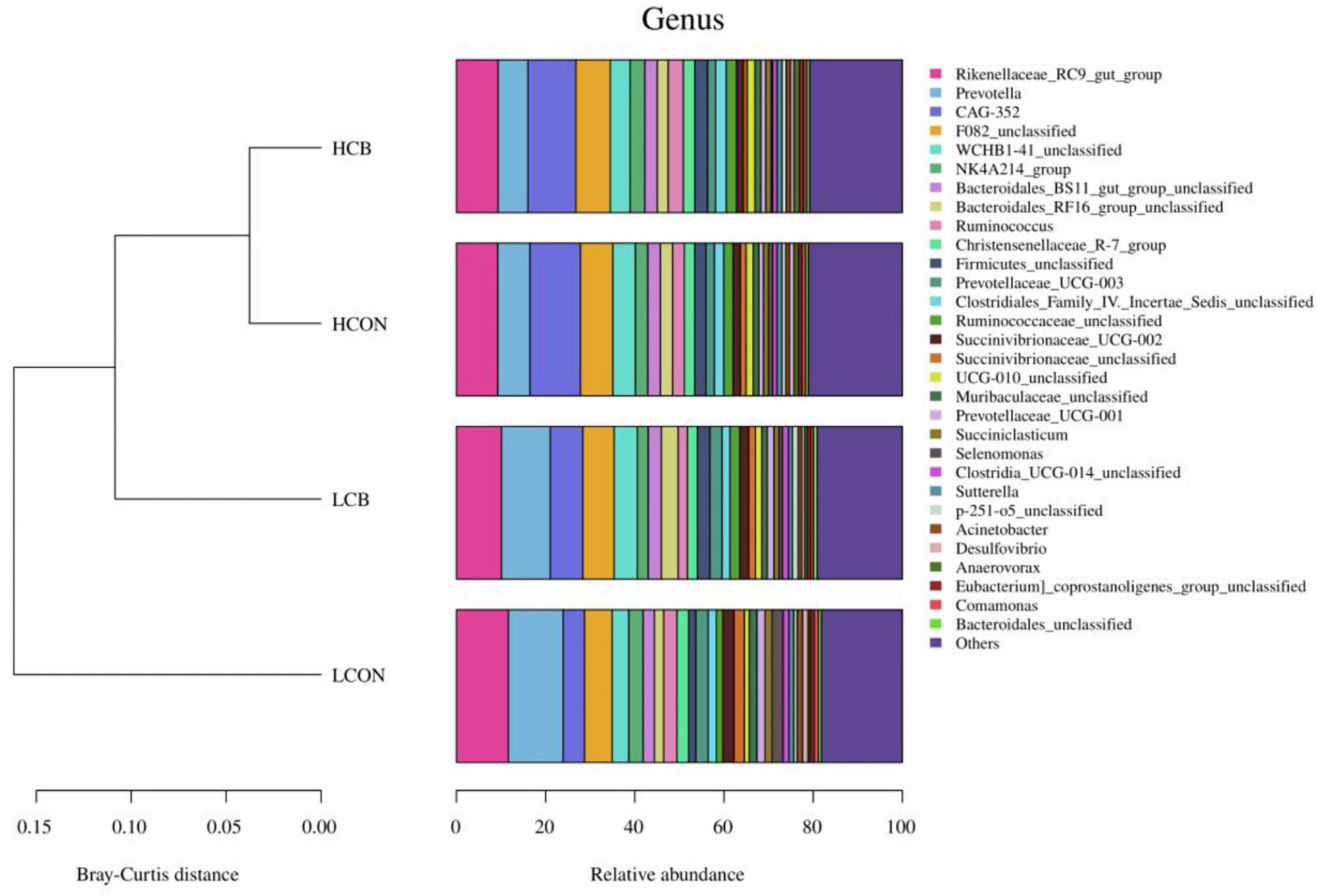
Top 30 relative abundances of the microbiota of ruminal fermentation fluid after 24 h of *in vitro* incubation that determined at the genus level (top 30). HCON, control at media pH 6.6; LCON, control at media pH 6.0; HCB, *C. butyricum* supplemented at 1 ×10^6^ CFU/bottle at media pH 6.6; LCB, *C. butyricum* supplemented at 1 ×10^6^ CFU/bottle at media pH 6.0.

**Figure 6:**
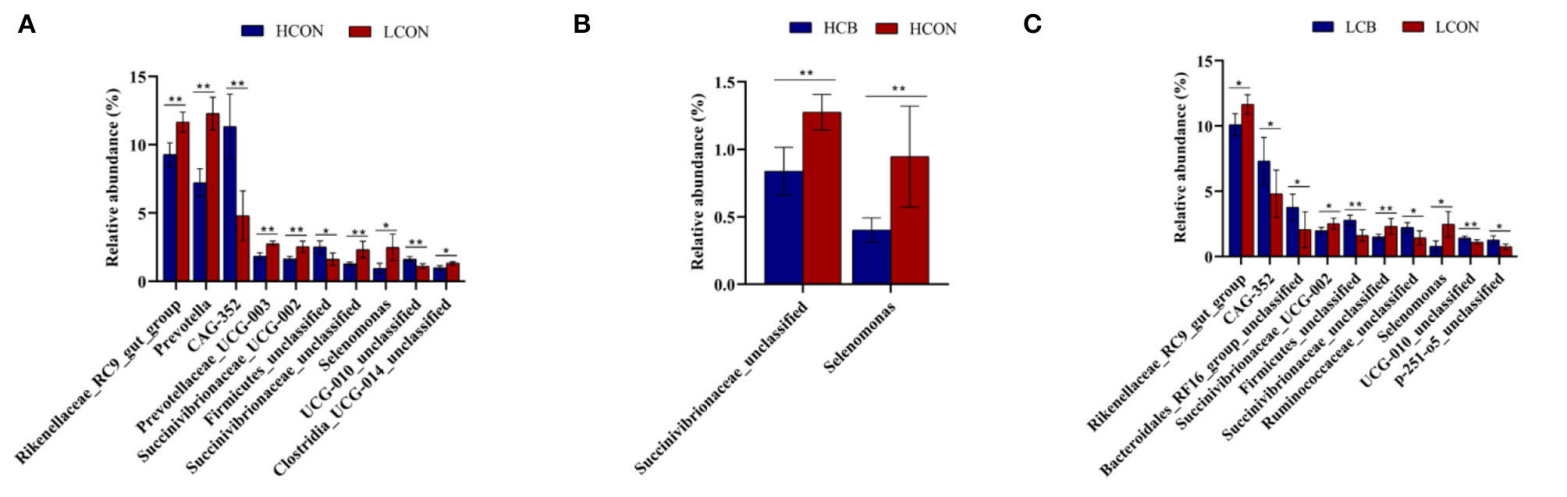
Top 10 relative abundance of microbiota in ruminal fermentation fluid that is significantly different between groups at the genus level. **(A)** HCON vs. LCON; **(B)** HCB vs. HCON; **(C)** LCB vs. LCON. HCON, control at media pH 6.6; LCON, control at media pH 6.0; HCB, *C. butyricum* supplemented at 1 ×10^6^ CFU/bottle at media pH 6.6; LCB, *C. butyricum* supplemented at 1 ×10^6^ CFU/bottle at media pH 6.0. Values are shown as means ± standard errors. *P <0.05; **P <0.01.

At the genus level, the top 30 RA of microbiota in fermentation fluid after 24 h of incubation were shown in Figure 5; Supplementary Figure S3. The top 10 alterations of RA of microbial communities at genus level were showed in Figure 6. The RA of *Rikenellaceae_RC9_gut_group* (*P* <0.01), *Prevotella* (*P* <0.01)*, Prevotellaceae_UCG-003* (*P* <0.01), *Succinivibrionaceae_UCG-002* (*P* <0.01), *Succinivibrionaceae_unclassified* (*P* <0.01), *Selenomonas* (*P* <0.05), and *Clostridia_UCG-014_unclassified* (*P* <0.05) were lower, whereas the RA of *CAG-352* (*P* <0.01), *Firmicutes_unclassified* (*P* <0.05), and *UCG-010_unclassified* (*P* <0.01) were greater at media pH 6.6 than pH 6.0 (Figure 6A). At media pH 6.6, the RA of *Succinivibrionaceae_unclassified* and *Selenomonas* decreased (*P* <0.01) with addition of *C. butyricum* compared with control (Figure 6B). At media pH 6.0, supplementation of *C. butyricum* decreased the RA of *Rikenellaceae_RC9_gut_group* (*P* <0.05), *Succinivibrionaceae_UCG-002* (*P* <0.05), *Succinivibrionaceae_unclassified* (*P* <0.01), and *Selenomonas* (*P* <0.05), and increased the RA of *CAG-352* (*P* <0.05), *Bacteroidales_RF16_group_unclassified* (*P* <0.05), *Firmicutes_unclassified* (*P* <0.01), *Ruminococcaceae_unclassified* (*P* <0.05), *UCG-010_unclassified* (*P* <0.01), and *p-251-o5_unclassified* (*P* <0.05) compared with control (Figure 6C).

## Discussion

### Gas Production, DMD, and Fermentation Characteristics

The study aimed to explore the effects of *C. butyricum* on *in vitro* rumen fermentation using a high grain substrate and whether the effects depend on the supplemental dose of *C. butyricum* and fermentation pH level. The high grain substrate used in the current study was a typical diet fed to lactating dairy cows. Ruminal pH is a key factor to affect the microbial population and fermentation end products. Moreover, the rumen pH varies substantially among individual animals or within an animal diurnally; it can be affected by various factors such as diet formulation and feed processing, in which below 6.0 was considered as a threshold value for subacute rumen acidosis (Russell and Wilson, 1996). In addition, the selected media pH of 6.0 and 6.6 in this study was also according to the low and high media pH used in a dual-flow continuous culture conducted by Colombatto et al. (2003). After 24 h of incubation, the final media pH was 6.11 and 6.72, respectively, which were close to our target media's low pH (6.0) and high pH (6.6), indicating high buffering of fermentation media despite fermentation acids produced from the high grain substrate in the current study. The greater GP, DMD, total VFA concentration, and ratio of A:P were consistently greater at media pH 6.6 vs. pH 6.0 in this study. This is in agreement with our previous studies that DMD, production of gas, total VFA concentration, and the ratio of A:P were greater for media pH 6.5 than pH 5.8 *in vitro* with either high forage or high grain substrate (Jiao et al., 2018, 2019). Similarly, Yang et al. (2002) reported that the DMD, concentrations of VFA, and the ratio of A:P linearly increased with increasing pH levels from 5.5 to 6.5. It is well-documented that the growth of fibrolytic bacteria is inhibited (Russell and Dombrowski, 1980) and that fiber digestion is impaired when fermenter pH falls below 6.2 (Hoover et al., 1984). Therefore, alternation of the fermentation pattern with increased A:P ratio indicated an improved fiber digestion at media pH 6.6 compared to pH 6.0.

In the current study, the quadratically changed GP kinetics and DMD (numerically) with increasing supplementation of *C. butyricum* at high media pH appeared to be consistent with the report by Cai et al. (2021) who found greater *in vitro* rumen digestibilities of DM and NDF with medium and medium-high doses of *C. butyricum* addition, but lower digestibilities of DM and NDF with high dose. The media pH was also higher with medium than high doses of *C. butyricum* in the study of Cai et al. (2021). These results suggest that high media pH would favor the activity of *C. butyricum* in the rumen, which might explain the positive effect of *C. butyricum* on GP and DMD at media pH 6.6 but not at media pH 6.0 in the present study. Adding *C. butyricum* consistently altered the VFA profiles, although total VFA concentration was not affected regardless of media pH levels. Our results are also in agreement with other findings. Li et al. (2021) reported that dietary supplementation of *C. butyricum* with 2 × 10^8^ CFU/kg DM increased the molar proportions of propionate and butyrate and decreased the proportion of acetate without changing the total VFA concentration in the rumen of heifers. Whereas, in the study of Cai et al. (2021), both total VFA concentration and VFA profiles were increased by supplementing *C. butyricum* at 0.05% and 0.10% of the basal diet; the increased VFA concentration was due to increased *in vitro* rumen DMD. It is reported that as a butyrate producer, *C. butyricum* could produce short-chain fatty acids and nutritional factors such as enzymes (exo-pectate lyase, pectin methylesterase, and endo-pectate lyase) and vitamins (vitamins B and E), which would favor the activities of both cellulolytic and amylolytic bacteria, thereby improving rumen fermentability (Araki et al., 2002; Li et al., 2021). This may explain the trend of a greater molar proportion of acetate with supplemented *C. butyricum* at a low dose (0.5 × 10^6^ CFU/bottle) than control at high media pH. The greater butyrate proportion with *C. butyricum* than control at low media pH or with medium and high (trend) dose of *C. butyricum* at high pH explained the production of butyrate by *C. butyricum*. The increased concentrations of NH_3_-N with supplementation of *C. butyricum* at either media pH 6.0 or pH 6.6 compared with control in the current study is consistent with Cai et al. (2021). Those authors found an increase in the rumen NH_3_-N concentration with supplemented *C. butyricum* both in *in vitro* and *in vivo*, suggesting that supplementation of *C. butyricum* enhanced the microbial proteolytic activity in the rumen. Nevertheless, the microbial protein synthesis in the rumen was not reported in that study (Cai et al., 2021).

### Rumen Bacterial Microbiota

The rumen function could be reflected by the diversity and richness of the rumen microbiome (Xie et al., 2018). The microbial diversity can be evaluated with alpha diversity including different indexes: OTU, Shannon, Simpson, Chao1, and Evenness. The OTU is defined as a collection of 16S rRNA sequences that have a certain percentage of sequence divergence. The Shannon index is an information statistic index assuming all species that are represented in a sample and that they are randomly sampled, while the evenness refers to the similarity in the abundances of different species in the microbial community. Whereas, the Simpson index is a dominance index to give more weight to common or dominant species. Finally, Chao1 is a non-parametric method for estimating the number of species in a community. This study showed the consistent interaction (significant or trend) between media pH level and dose of *C. butyricum* supplementation that increased the number of OTUs, indexes of Shannon, Simpson, Chao1, and Evenness at media pH 6.0, but no effects of *C. butyricum* at pH 6.6. These results suggest that the supplementation of *C. butyricum* may improve rumen microbiota at low fermentation pH where ruminal microbial activities are risked to be compromised (Juan et al., 2017).

The PCoA plotting based on unweighted Unifrac distances reflected significant differences between groups of control at media pH 6.0 and pH 6.6, and between groups of control and *C. butyricum* supplementation at media pH 6.0. Similarly, the Venn diagram revealed that the diversity of microbiota was reduced, and the diversity of microbiota was increased by supplementation of *C. butyricum* at low media pH. It is a general consensus that the rumen pH would be reduced with increasing grain inclusion in the diets of ruminants because some gram-negative bacteria would be decreased in a low-pH environment (Khafipour et al., 2009). Trabi et al. (2020) reported that the diversity indices of ruminal epithelium microbiome decreased when sheep were fed a high-grain vs. low-grain diet, which is consistent with the current found. However, the increased diversity indices with supplemented *C. butyricum* at low media pH may be attributed to relieving microbiota which was inhibited by the low media pH.

Several studies showed that *Bacteroidota* and *Firmicutes* are the dominant phyla existing within the rumen ecosystem (Amat et al., 2021; Hinsu et al., 2021; Virgínio Júnior et al., 2021). Consistently, the present study confirmed that *Bacteroidota* and *Firmicutes* are the top two predominant microbes regardless of media pH level or *C. butyricum* supplementation. The *Firmicutes* were sensitive to low pH (Ebeid et al., 2020), resulting in an increased ratio of *Firmicutes* to *Bacteroidetes* at media pH 6.6 than pH 6.0. It was reported that the *Firmicutes* to *Bacteroidetes* ratio is closely associated with adipogenesis metabolism and degradation of structural polysaccharides (Jami et al., 2013; Zened et al., 2013). Therefore, the higher DMD and total VFA concentration could be explained by the improvement or degradation of structural polysaccharides due to the higher ratio of *Firmicutes* to *Bacteroidetes* with high media pH in this study. Moreover, the ratio of *Firmicutes* to *Bacteroidetes* indicates the proportion of different VFA profiles in rumen such as propionate produced by *Bacteroidetes* (Paoli et al., 2019), which is consistent with the higher A:P ratio at media pH 6.6 than pH 6.0. Previous studies have demonstrated that *C. butyricum* could modulate the microbiota in the intestine of broiler chickens (Molnar et al., 2020), feces of weaned piglets (Zhang et al., 2018), or rumen of heifers (Li et al., 2021). Auffret et al. (2017) reported that a high ratio of *Proteobacteria* to (*Firmicutes* + *Bacteroidetes*) was a good indicator for rumen dysbiosis. Thus, the decreased relative abundance of *Proteobacteria* with supplemented *C. butyricum* at either media pH levels in the current study indicated an improvement of microbial stability in the rumen.

At the genus level, the relative abundances of the top three, *Rikenellaceae_RC9_gut_group, CAG-352*, and *Prevotella*, were all changed by media pH. The genus *Rikenellaceae_RC9_gut_group* belongs to the family *Rikenellaceae*, which plays a role in the fermentation of carbohydrates or proteins (Su et al., 2014; Yan et al., 2018). Qiu et al. (2022) found that its relative abundance was positively correlated with rumen pH, dietary fiber, and acetate to propionate ratio. However, the greater relative abundance of *Rikenellaceae_RC9_gut_group* was observed at high media pH in the present study and the inconsistent results may be attributed to the different animal species (dairy vs. beef cattle). The genus *Prevotella* has been defined as a metabolically and genetically diverse bacterial population, which was involved in the degradation of cellulose, hemicellulose, and hydrolysis of starch, protein, and peptides (Dai et al., 2015; Wang et al., 2020). The higher molar proportion of propionate at media pH 6.0 than pH 6.6 could be explained by a greater relative abundance of *Prevotella* which are reported to be propionate producers. Moreover, the greater relative abundance of *CAG-352* at high media pH was noticed, but the role of *CAG-352* during rumen fermentation is still limited. A recent study demonstrated a negative correlation between the relative abundance of *CAG-352* with serum concentration of glucose and a positive correlation with serum concentrations of creatinine, total protein, albumin, alkaline phosphatase, and non-esterified fatty acid in cattle (Qiu et al., 2022). In addition, the changes in relative abundances of several species at phylum and genus levels with supplementation of *C. butyricum* at both media pH levels in the current study may be contributed by the nutritional factors (enzymes and vitamins) produced by *C. butyricum*. The RA of unclassified *Succinivibrionaceae* and *Selenomonas* was positively correlated with propionate in the rumen (Sawanon et al., 2011; Xue et al., 2019). Therefore, the quadratic decrease of the molar proportion of propionate may be supported by the decrease in RA of *Succinivibrionaceae_unclassified* and *Selenomonas* with supplementation of *C. butyricum* at either media pH 6.6 or pH 6.0. It is worth noticing that more species of bacteria were affected by *C. butyricum* supplementation at media pH 6.0 than pH 6.6 either at phylum or genus level in this study, indicating a greater activity of *C. butyricum* at low media pH. To our best knowledge, little work has been done on the regulatory mechanism of C. *butyricum* in rumen fermentation varying with media pH levels.

## Conclusion

The greater GP, DMD, total VFA concentration, and A:P ratio at media pH 6.6 than 6.0 was suggested to be partly attributed to the alternation of relative abundances of microbiota such as *Firmicutes* and *Bacteroidetes* at the phylum level. This quadratically changed the proportions of propionate and butyrate, and the NH_3_-N concentration with increasing supplementation of *C. butyricum* indicated a dose-dependent manner regardless of media pH levels. Furthermore, the supplementation of *C. butyricum* increased the rumen microbiome diversity at media pH 6.0 and altered the relative abundances of microbiota at either phylum or genus level at both low and high media pH. These results elucidated that supplementation of *C. butyricum* had a consistent impact on microbiota at the low media pH of 6.0. Further study is warranted to investigate the effects of *C. butyricum* on rumen microbiota at lower rumen pH because the rumen pH is often below 6.0 in high-producing ruminants fed high-grain diets.

## Data Availability Statement

The datasets presented in this study can be found in online repositories. The names of the repository/repositories and accession number(s) can be found here: NCBI BioProject - PRJNA823901.

## Ethics Statement

The animal study was reviewed and approved by the Animal Care Committee, College of Animal Science and Technology, and Northeast Agricultural University.

## Author Contributions

PJ and XX conceived and designed the experiments. PJ, ZW, XW, YZ, and YY performed the experiments. GH, CL, and LW analyzed the data. PJ and WY contributed to the writing of the manuscript. All authors reviewed and approved the manuscript.

## Funding

This research was supported by the Natural Science Foundation of Heilongjiang Province (YQ2021C018), the Postdoctoral Foundation of Heilongjiang Province (LBH-Z21100), and the Young Talents Project of Northeast Agricultural University (19QC42).

## Conflict of Interest

LW was employed by Hubei Greensnow Biological Technology Co., Ltd. The remaining authors declare that the research was conducted in the absence of any commercial or financial relationships that could be construed as a potential conflict of interest.

## Publisher's Note

All claims expressed in this article are solely those of the authors and do not necessarily represent those of their affiliated organizations, or those of the publisher, the editors and the reviewers. Any product that may be evaluated in this article, or claim that may be made by its manufacturer, is not guaranteed or endorsed by the publisher.
