# Supplementary figures and images for: Effect of Clostridium butyricum Supplementation on in vitro Rumen Fermentation and Microbiota With High Grain Substrate Varying With Media pH Levels
Source: Front Microbiol. 2022 Jun 23;13:912042. doi: 10.3389/fmicb.2022.912042 (PMC9260501; doi:10.3389/fmicb.2022.912042)

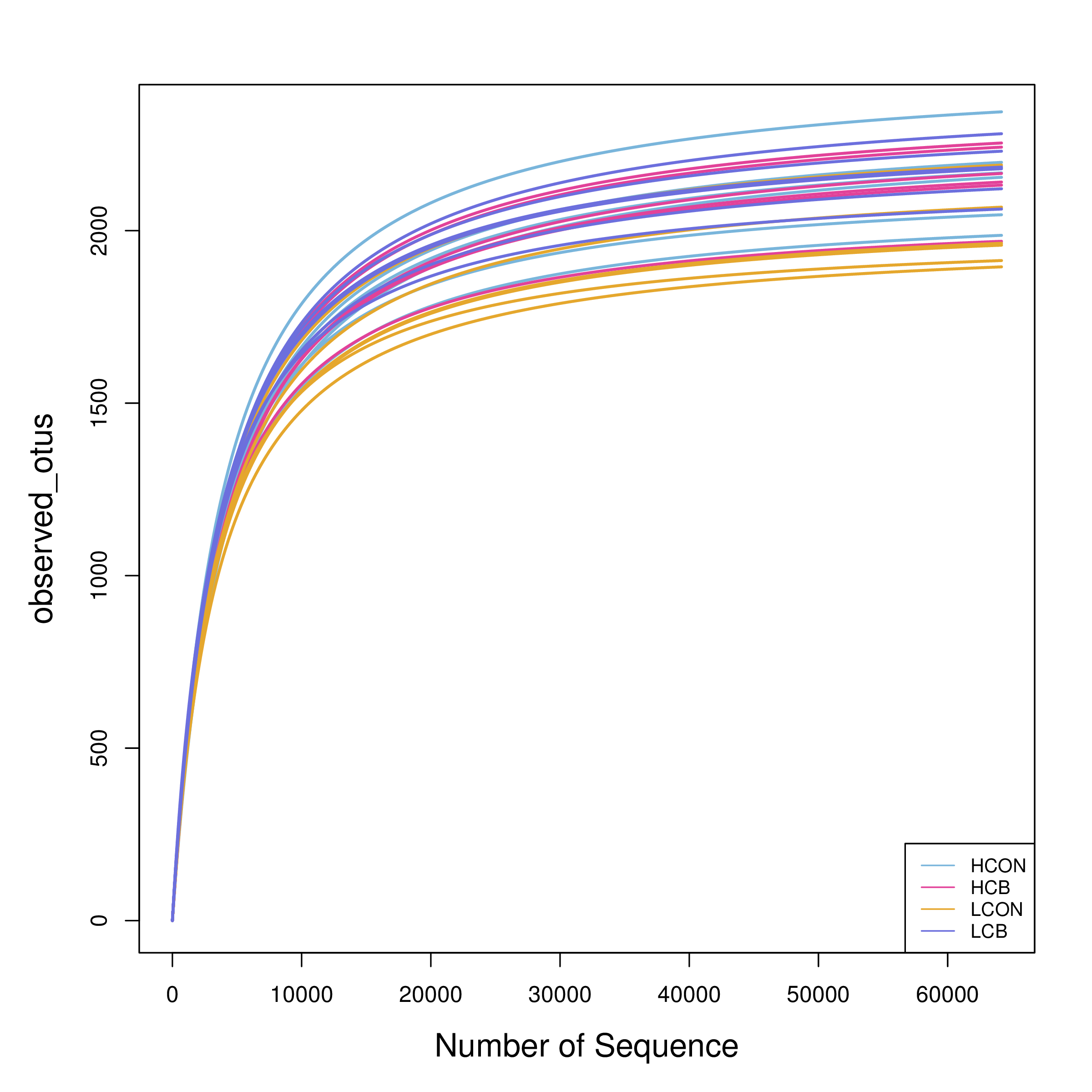

Supplement: Supplementary file 1 [file Image_1.PNG]

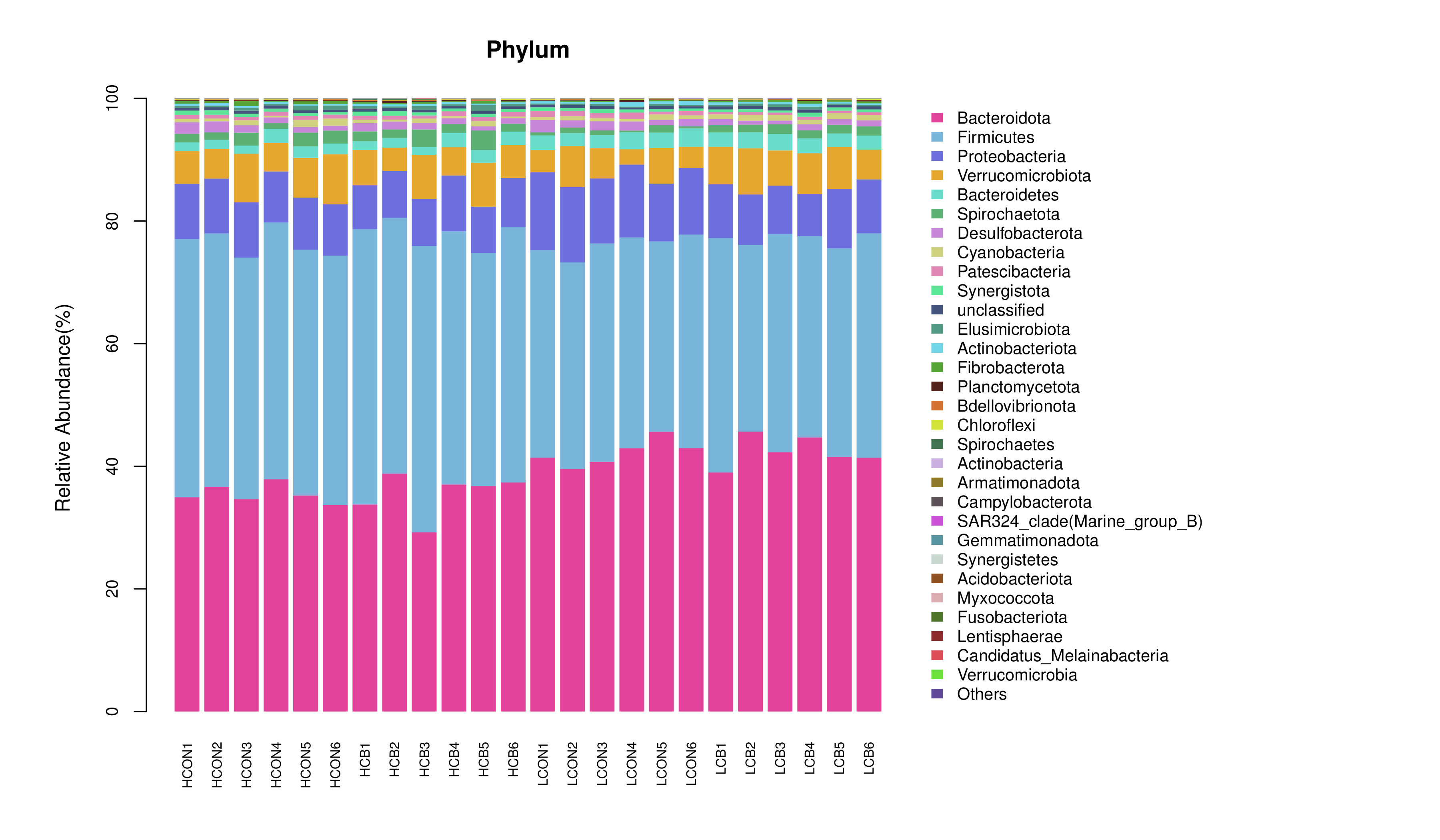

Supplement: Supplementary file 2 [file Image_2.PNG]

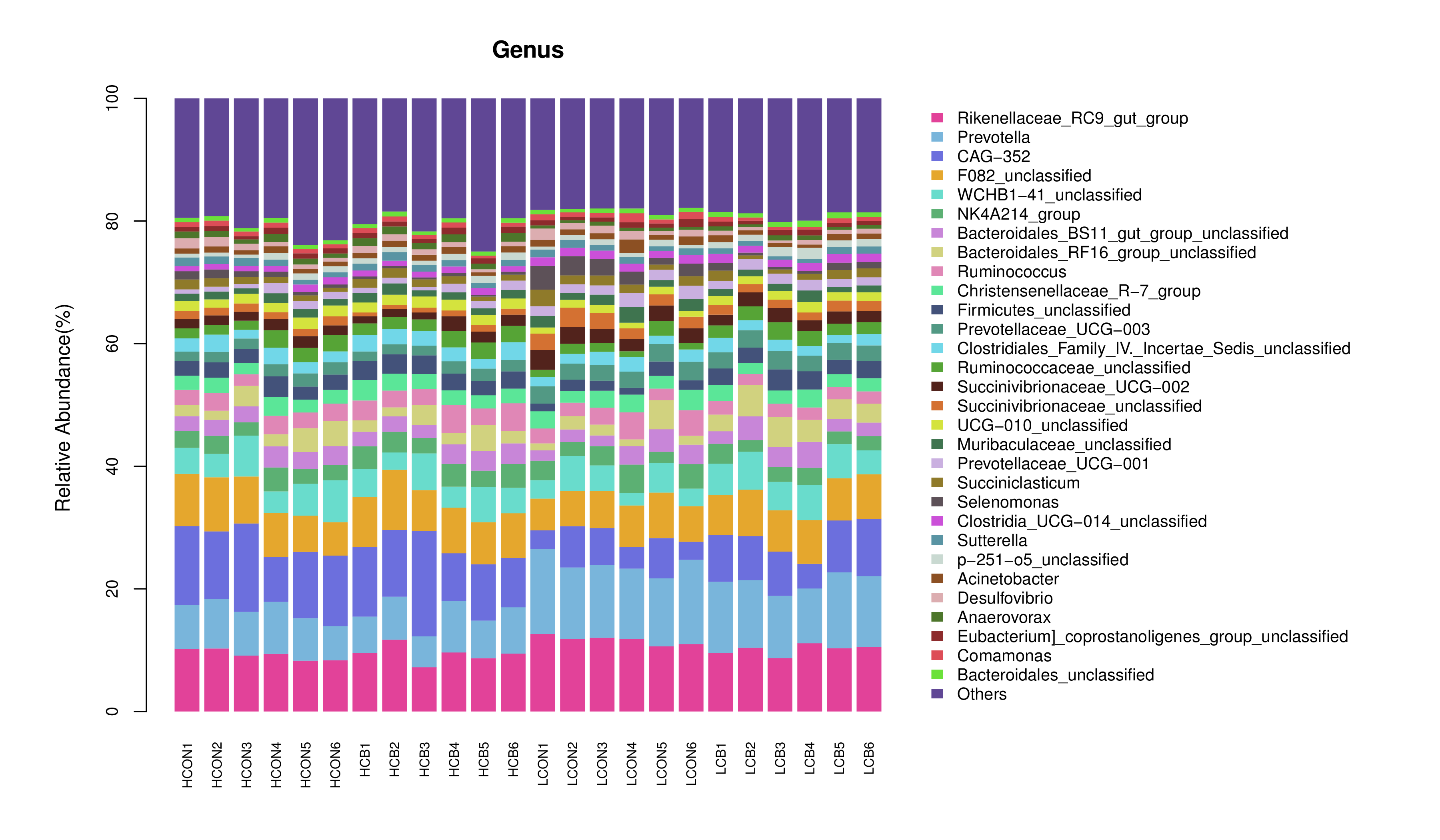

Supplement: Supplementary file 3 [file Image_3.PNG]
